# Design of a Broadband Tunable Terahertz Metamaterial Absorber Based on Complementary Structural Graphene

**DOI:** 10.3390/ma11040540

**Published:** 2018-03-31

**Authors:** Mu Lin Huang, Yong Zhi Cheng, Zheng Ze Cheng, Hao Ran Chen, Xue Song Mao, Rong Zhou Gong

**Affiliations:** 1 Engineering Research Center for Metallurgical Automation and Detecting Technology Ministry of Education, Wuhan University of Science and Technology, Wuhan 430083, China; hml_q1@163.com (M.L.H.); 18627140931@163.com (H.R.C.); xsmao@wust.edu.cn (X.S.M.); 2 School of Electronic and Information Engineering, Hubei University of Science and Technology, Xianning 437100, China; czz8986@126.com; 3 School of Optical and Electronic Information, Huazhong University of Science and Technology, Wuhan 430074, China

**Keywords:** terahertz metamaterial absorber, graphene, broadband tunable absorption, surface plasmon resonances

## Abstract

We present a simple design for a broadband tunable terahertz (THz) metamaterial absorber (MMA) consisting of a complementary cross-oval-shaped graphene (CCOSG) structure and dielectric substrate placed on a continuous metal film. Both numerical simulation and theoretical calculation results indicate that the absorbance is greater than 80% from 1.2 to 1.8 THz, and the corresponding relative bandwidth is up to 40%. Simulated electric field and power loss density distributions reveal that the broadband absorption mainly originates from the excitation of continuous surface plasmon resonance (SPR) on the CCOSG. In addition, the MMA is polarization-insensitive for both transverse-electric (TE) and transverse-magnetic (TM) modes due to the geometry rotational symmetry of the unit-cell structure. Furthermore, the broadband absorption properties of the designed MMA can be effectively tunable by varying the geometric parameters of the unit-cell and chemical potential of graphene. Our results may find promising applications in sensing, detecting, and optoelectronic-related devices.

## 1. Introduction

Metamaterial absorbers (MMAs) as the engineered sub-wavelength periodic structures or functional materials have recently attracted tremendous attention in terahertz (THz) spectroscopy, since THz electromagnetic (EM) waves can be successfully absorbed by few natural materials [1,2,3,4,5,6,7,8,9,10,11,12,13,14,15,16,17,18]. Due to extensive applications in sensing [19], detection [20], modulation [21], thermal emission [22], and so on, a variety of THz MMAs have been proposed and investigated. However, most traditional MMAs have some drawbacks in that the absorption level and working frequency are uncontrolled apart from changing the geometrical parameters of the unit-cell structure, which will limit their practical applications [1,2,3,4,5,6,7,8,9,10,11,12,13,14]. Nowadays, to achieve tunable properties of the MMAs, some effective methods have been proposed and demonstrated, such as machine [23], laser [24,25,26], temperature [27,28], and voltage modulation [29,30,31,32,33,34,35]. However, the realization of the broadband tunable and strong absorption of the MMA is still a great challenge. Thus, it is still highly desirable to explore new modulation methods and design tunable MMAs with high performance.

Graphene, as a novel mono-layer two-dimensional material consisting of carbon atoms array, has the favorable electric characteristic that the conductivity depends on chemical potential (*μ*_c_) being continuously tuned by chemical doping and bias voltage [36,37]. This unique characteristic makes graphene a desirable candidate to design tunable MMA. Based on effective media theory, the complex permittivity *ε*(*ω*) and permeability *μ*(*ω*) of these MMAs can be changed when the conductivity of graphene sheet is effectively controlled by gate voltage or chemical doping [24]. Recently, some MMA-based-graphene have been proposed, which can achieve strong and tunable absorption by changing the chemical potential (*μ*_c_) [30,31,32,33,34,35,38,39,40,41,42,43,44,45]. According to the well-known Kubo formula, the real part of permittivity of a graphene sheet is negative from THz to optical frequencies [30]. This means that the strong surface plasmons (SPs) can be sustained, propagating along the sheet in our interesting frequency region [37,46]. In addition, SPRs will be excited when the value of the wave vector (*k*_G_) in graphene is close to the incident EM wave vector (*k*_0_) [38]. Surface plasmons resonances (SPRs) could be effectively excited in a graphene sheet as that is one key to obtaining strong absorption properties [31,32,38,39]. Zhu et al. proposed a MMA using a periodic array of graphene ribbons to achieve the tunability of absorption peak frequency in THz region [31]. However, it worked only in a narrow band range. Gao et al. presented a tunable broadband THz absorber with a graphene sheet sandwiched between periodical arrays of dielectric bricks and dielectric substrate [38]. Xiao et al. designed a multilayer cross-shaped graphene structure MMA that can achieve tunable broadband absorption in the THz region [39]. Nevertheless, the above mentioned multiband or broadband THz MMAs based on structural or patterned graphene are usually discontinuous or multilayer, which causes complexity of fabrication in practical applications. In addition, it is difficult to tune the conductivity of graphene via extra bias voltage [35,39,41].

In this work, we proposed a simple design for a broadband tunable MMA based on a periodical single-layered complementary cross-oval-shaped graphene (CCOSG) structure in the THz region. The simulation results indicate that the designed MMA can achieve strong absorption (over 80%) from 1.2 to 1.8 THz when *μ*_c_ is 0.5 eV. The designed MMA based on structured graphene could significantly enhance the absorption level compared with one using a single-layer graphene sheet [30]. In addition, the MMA is polarization-insensitive and the absorption of full bandwidth at half maximum (FWFH) could be shifted from 0.92–1.68 THz to 1.20–1.96 THz when *μ*_c_ is increased from 0.3 to 0.7 eV.

## 2. Structure Design, Theory, and Simulation

**Figure 1:**
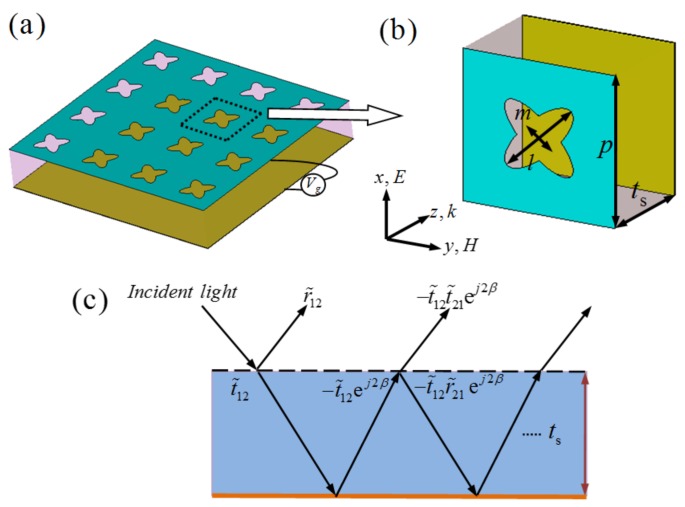
The scheme of designed THz MMA: (**a**) the period array and (**b**) unit-cell structure; (**c**) the interference model.

Figure 1a,b shows the design scheme of MMA composed of a single-layer periodical CCOSG sheet deposited on a loss-free dielectric substrate (SiO_2_) with a continuous metal (Cu) film on the bottom. As shown in Figure 1a,b, the period of unit-cell structure is *p* = 50 μm. The long and short radius of the complementary oval structure are *l* = 30 μm and *m* = 11 μm, respectively. The loss-free SiO_2_ with the thickness of *t*_s_ = 34 μm and relative permittivity of *ε*_r_ = 3.9 is selected as the middle dielectric substrate. The thickness and conductivity of bottom copper film are *t*_m_ = 0.5 μm and *σ* = 5.8 × 10^7^ S/m, respectively. Similar to previous research [45], we assume an effective thickness *t*_g_ = 1 nm for a graphene sheet consisting of a mono-layer carbon atoms array in our design.

Furthermore, as shown in Figure 1a, the three-layer structure of the MMA is similar to a Fabry–Pérot resonance cavity, which can induce destructive and constructive interferences and finally result in multiple reflections and transmissions for the incident THz waves. Here, we can adopt the multiple interference theory analyze the absorption properties. As shown in Figure 1a, the multi-reflection interference model contains two interfaces: air–spacer with periodical CCOSG array, and spacer–substrate. In this design, the near-field coupling effect between the CCOSG sheet and ground plane can be neglected, since the thickness of the dielectric substrate is large enough. As shown in Figure 1c, when the incident THz wave enters the MMA, part is reflected at the CCOSG–air interface, and the other part emits from the interface after multiple reflections and transmissions. Thus, the overall reflection coefficient of the structure is expressed as follows [14]:

(1) $${\tilde{r}}_{all}={\tilde{r}}_{12}-\frac{{\tilde{t}}_{12}{\tilde{t}}_{21}e^{j2\beta }}{1+{\tilde{r}}_{21}e^{j2\beta }},$$

where *β* = *nk*_0_*t*_s_ is the propagation phase, *n* and *t*_s_ are the refractive index and thickness of dielectric substrate SiO_2_, respectively, and *k*_0_ is the free space wave number. In addition, ${\tilde{r}}_{12}$ and ${\tilde{t}}_{12}$ represent the reflection and transmission coefficient at the air–CCOSG interface, respectively. At the CCOSG–substrate interface, the corresponding reflection and transmission coefficients can be defined by ${\tilde{r}}_{21}$ and ${\tilde{t}}_{21}$, respectively. Therefore, the total absorbance can be derived by *A*(*ω*) = 1 − |${\tilde{r}}_{all}$|^2^.

To realize the tunability of the proposed MMA, we added the external bias voltage to the MMA, as shown in Figure 1a. Based on previous studies [32,47], the chemical potential (*μ*_c_) could be controlled by bias voltage:

(2) $$\mu_{c}=\hbar v_{f}\sqrt{\frac{\pi \varepsilon_{0}\varepsilon_{r}V_{g}}{et_{s}}},$$

where *v*_f_ is Fermi velocity, *ε*_0_ is the vacuum permittivity constant, *e* is an electron charge constant, *ћ* is the reduced Planck’s constant, *μ*_c_ is chemical potential, and *V*_g_ is the extra bias voltage. We assume that the working frequency range is 0.5–2 THz, and the chemical potential of the graphene sheet is 0.3 to 0.7 eV [38]. According to the well-known Kubo formula, the conductivity model of graphene sheet can be simplified as follows [37,48]:

(3) $$\sigma_{g}\approx j\frac{e^{2}K_{B}T}{\pi \hbar^{2}(\omega +j\tau^{-1})}(\frac{\mu_{c}}{K_{B}T}+2\ln(e^{\left(-\frac{\mu_{c}}{K_{B}T}\right)}+1)),$$

where *ω* is radio frequency, *T* is environment temperature, *τ* is relaxation time, and *K_B_* is Boltzmann’s constant. The other parameters have been defined previously. In our work, *T* is fixed at 300 K and *τ* is assumed to be 0.5 ps. Thus, the relative permittivity of graphene can be obtained by *ε*_g_ = 1 + *jσ*_g_/*ωε*_0_*t*_g_ [35].

To study the efficiency of the proposed MMA, the CST microwave studio based on finite integration technique (FIT) is employed to perform full-wave simulation. In the simulation, the periodic boundary conditions are used in the *x*-*y* plane and incident THz waves propagate along the *z*-direction, as shown in Figure 1a,b. The electric and magnetic components of the incident THz wave are along the *x* and *y* direction, respectively, and the wave vector *k* is along the *z*-direction, as depicted in Figure 1b. The absorbance is calculated by *A*(*ω*) = 1 − *R*(*ω*) − *T*(*ω*), where *R*(*ω*) and *T*(*ω*) are the reflectance and transmittance, respectively. Because the thickness of the bottom continuous copper film is much greater than skin depth in our frequency range of interest, the incident light is prevented through the whole MMA and the transmittance can be ignored. Thus, the absorbance can be rewritten as *A*(*ω*) = 1 − *R*(*ω*).

## 3. Results and Discussion

**Figure 2:**
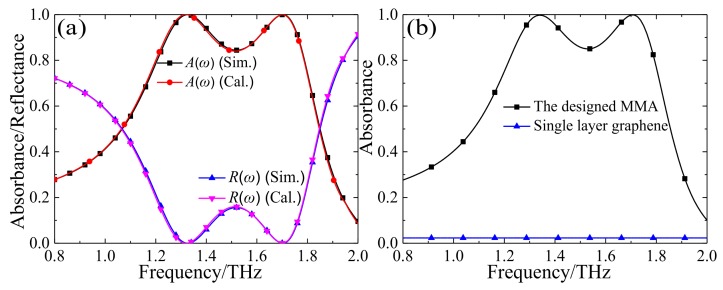
The numerical results of the proposed MMA with *μ*_c_ = 0.5 eV: (**a**) the simulated and calculated reflectance and absorbance of the designed MMA based on CCOSG sheet; (**b**) the simulated absorbance of the MMA based on CCOSG sheet and bare mono-layer graphene.

Figure 2 shows the numerical results of the proposed MMA when the chemical potential *μ*_c_ is set to 0.5 eV. As depicted in Figure 2a, the simulated reflectance is below 20% from 1.2 to 1.8 THz, and the corresponding absorbance is greater than 80%. Thus, the relative stronger absorption bandwidth is up to 40%. In addition, at two resonance frequencies (*f*_1_ = 1.34 THz and *f*_2_ = 1.71 THz), the absorption levels are both greater than 99.9%. Obviously, the simulation results agree well with the calculation ones based on interference theory. Figure 2b shows the absorbance of the MMA based on the CCOSG structure and the single-layer continuous graphene sheet, respectively. It can be seen that the absorption level of the single-layer continuous graphene sheet is very small (about only 2.3%), and far less than that of the MMA based on the CCOSG structure. Thus, the high absorption level of the MMA with CCOSG structure design can be significantly enhanced for the incident THz waves [49]. 

**Figure 3:**
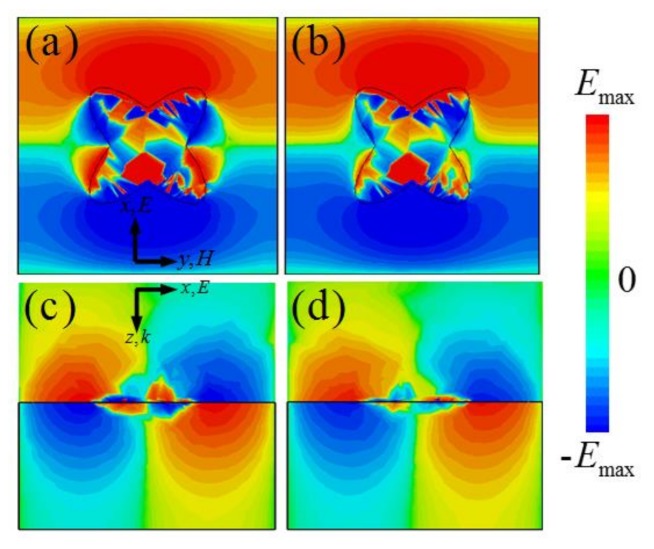
The electric field (*E*_z_) distributions of the *x*–*y* and *x*–*z* planes of the unit-cell structure at (**a**,**c**) *f*_1_ = 1.34 THz and (**b**,**d**) *f*_2_ = 1.71 THz, respectively (*μ*_c_ = 0.5 eV).

To better understand the physical mechanism of the stronger absorption behavior observed in the broadband, the electric field (*E*_z_) distributions of the *x*–*y* and *x*–*z* planes of the unit-cell structure at two resonance frequencies are investigated numerically. Figure 3a,b shows that the electric field (*E*_z_) distributions mainly concentrate on the four corners of the CCOSG structure and the upper and lower sides of unit-cell structure. As shown in Figure 3c,d, surface plasmon polaritons (SPPs) are effectively excited at two absorption peak frequencies (*f*_1_ = 1.34 THz, *f*_2_ = 1.71 THz). Since strong absorption is obtained, *k*_G_ is about equal to *k*_0_ and SPRs are excited between *f*_1_ and *f*_2_. In addition, it can be observed that fundamental and second-order SPR are effectively excited at two resonance frequencies. Furthermore, since the dielectric substrate is loss-free and incident light is less absorbed by a single-layer graphene sheet in a broadband range, it can be conjectured that the strong SPRs noticeably enhance the absorption efficiency of the CCOSG structure of this MMA. However, there is a small difference in the field distributions for two resonance frequencies. Compared with the first resonance frequency, the fundamental SPR is slightly suppressed by the second-order one at the second resonance frequency.

**Figure 4:**
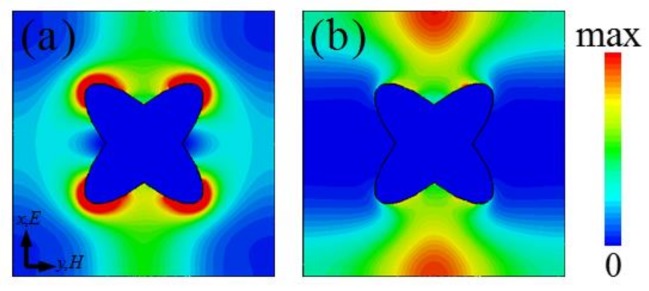
The loss density distributions of the *x*–*y* plane on the CCOSG surface: (**a**) *f*_1_ = 1.34 THz and (**b**) *f*_2_ = 1.71 THz, respectively (*μ*_c_ = 0.5 eV).

To further understand the absorption mechanism, we give the power loss density distributions of the *x*–*y* plane on the CCOSG surface at two absorption peak frequencies when *μ*_c_ = 0.5 eV, as shown in Figure 4. For the first absorption peak, as shown in Figure 4a, the THz energy is mainly dissipated at the four corners of the CCOSG structure, meaning that the absorption is principally based on fundamental SPR. However, as shown in Figure 4b, there are power losses at the upper and lower edges of the unit-cell structure at the second resonance frequency. The absorption mainly originates from excitation of second-order SPR at the second absorption peak. Thus, the loss density distributions further illustrate that the broadband absorption of this designed MMA is based on effective excitation of the fundamental and second-order SPRs. 

**Figure 5:**
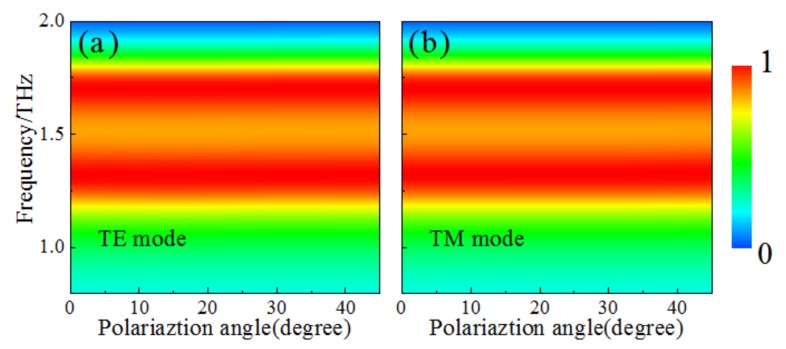
The absorption spectra under different polarization angles for (**a**) TE and (**b**) TM modes.

Polarization insensitivity is of considerable merit for MMAs in practical applications. Thus, it should be considered for our design under different polarization angles for both TE and TM modes. Owing to the rotation symmetry of the unit-cell structure, the absorption spectra of the designed MMA are basically unchanged when the polarization angle is from 0 to 45° for both TE and TM modes, as shown in Figure 5a,b.

**Figure 6:**
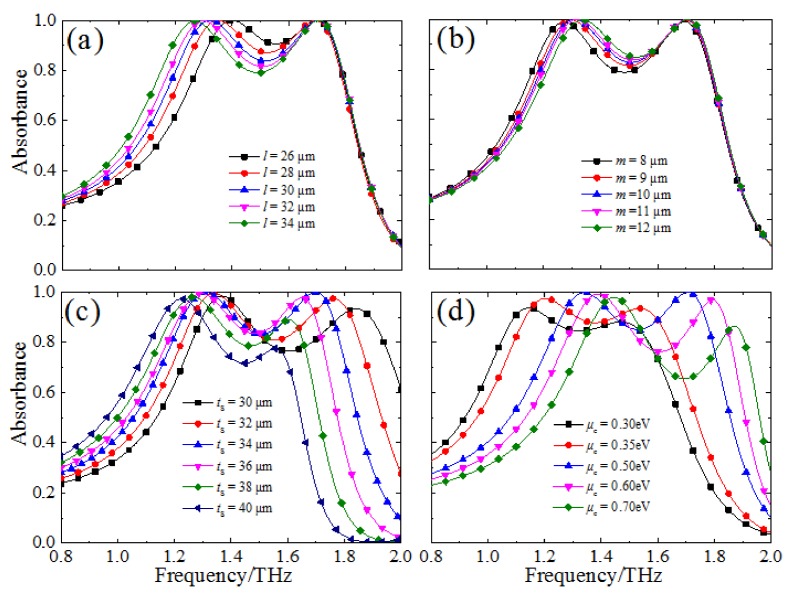
The absorption spectra under different geometric parameters of the unit-cell structure and chemical potential: (**a**,**b**) the long (*l*) and short (*m*) radius of the CCOSG structure; (**c**) the thickness (*t*_s_) of the dielectric substrate; (**d**) the chemical potential (*μ*_c_).

Taking things a step further, we also study the resonance absorption properties of the designed MMA with different geometric parameters for the unit-cell structure and chemical potential (*μ*_c_). As shown in Figure 6a, when the long radius (*l*) of the CCOSG structure increases from 26 to 34 μm, the strong absorption bandwidth is extended and the absorption level at the central frequency is gradually decreased. In addition, the first absorption is red-shifted from 1.4 to 1.27 THz and the second absorption peak is basically unchanged. Meanwhile, the proposed MMA maintains perfect absorption at two resonance frequencies. However, the case is much different for the short radius (*m*) of the CCOSG structure, as shown in Figure 6b. When *m* increases from 8 to 12 μm, the strong absorption bandwidth becomes narrower and the absorption level gradually increases at the central absorption frequency. In addition, the first absorption peak frequency is slightly blue-shifted from 1.27 to 1.34 THz, but the second is nearly unchanged. Similarly, the absorption level also achieves 99% at two resonance frequencies. It should be noticed that only the first resonance frequency is affected by changing *l* and *m*, since the incident THz wave energy is mainly dissipated at the four corners of the CCOSG structure at the first resonance frequency (see Figure 4a). Considering that the first SPR is localized at the four corners, the dispersion relation can be approximated for the localized SPR, which is matched with the simulation results [46]. The second SPR, which is excited in the graphene sheet with no gap, is nearly unchanged with different *l* or *m*. Based on SPRs in unstructured graphene sheets, the second SPR frequency is relative to *μ*_c_ [38]. Thus, the first resonance frequency and bandwidth are dynamically modulated by tuning the *l* or *m* value of the CCOSG structure.

Then, as shown in Figure 6c, when the thickness (*t*_s_) of the dielectric substrate increases from 30 to 40 μm, the frequency range of strong absorption is decreased and gradually red-shifted. In this case, two resonance absorption frequencies decrease gradually from 1.37 to 1.22 THz, and from 1.84 to 1.56 THz, respectively. Due to the increasing thickness of the resonant cavity, the working frequency range is gradually red-shifted, resulting in the resonance frequencies first being close to the central strong SPR range and then deviating from it [50]. Thus, the absorption level first increases and then decreases gradually with the increase in *t*_s_, and is at a maximum when *t*_s_ = 34 μm.

Finally, the absorption properties of the proposed MMA are further discussed when the chemical potential is tuned from 0.3 to 0.7 eV through external bias voltage, as shown in Figure 6d. The frequency range of the FWHM of the designed MMA is obviously blue-shifted from 0.92–1.68 THz to 1.20–1.96 THz when *μ*_c_ is increased from 0.3 to 0.7 eV. According to Equation (3), the working frequency band is blue-shifted to compensate for the increased conductivity of the graphene sheet [41]. When the chemical potential is 0.3 eV, the designed MMA displays great absorption at 1.15 THz and 1.47 THz. When *μ*_c_ increases from 0.3 to 0.5 eV, the absorbance of MMA is gradually enhanced and finally reaches 99.9% at 1.34 THz and 1.71 THz. For higher *μ*_c_, more charge carriers are needed to better excite SPRs for the normal incident THz waves. In addition, the blue-shifted frequency also contributes to inducing SPRs [51,52]. However, the absorption level will decrease gradually when further increasing *μ*_c_ (>0.5 eV). When *μ*_c_ = 0.7 eV, the two resonance frequencies are shifted to 1.45 THz and 1.87 THz, and the corresponding absorbance is decreased to 97.8% and 86.38%, respectively. Since the resonance frequencies gradually deviate from the central frequency range of the strong SPR, the absorption level decreases accordingly [50]. Moreover, at the central absorption frequency, the absorption level first increases slightly when *μ*_c_ increases from 0.3 to 0.35 eV. When *μ*_c_ increases further from 0.4 eV, the absorption level of the second frequency decreases and the strong absorption bandwidth gradually broadens. Overall, the designed MMA can achieve dynamically tunable absorption by adjusting the chemical potential, which has great potential applications in THz range.

## 4. Conclusions

In conclusion, we have proposed a broadband tunable MMA using a CCOSG structure and a dielectric substrate placed on a metallic film in the THz region. Numerical simulation results indicate that the proposed MMA can achieve high absorption of over 80% in the frequency range of 1.2–1.8 THz, and the corresponding relative bandwidth is up to 40%, which agrees well with calculated ones based on interference theory. The distributions of electric field and power loss density of the unit-cell structure reveal that the enhancement of absorption of MMA mainly originates from the effective excitations of fundamental and second-order SPRs on the CCOSG structure. In addition, the polarization-insensitive properties of the MMA are demonstrated by a numerical simulation. Furthermore, we discussed the influence of the geometrical parameters of the unit-cell structure and external bias voltage on the absorption properties of the MMA. Compared with conventional discontinuous or multi-layered graphene structures, the proposed high-performance MMA is easily fabricated and modulated. Due to its favorable performance, the proposed tunable THz MMA could find some potential applications in thermal imaging, thermal bolometers, biosensors, and so on.
